# ADVERSE REACTION TO ANESTHESIA IN A M.8993T>C CARRIER WITH LEIGH SYNDROME

**DOI:** 10.1590/1984-0462/;2019;37;1;00020

**Published:** 2019

**Authors:** Josef Finsterer

**Affiliations:** a University of Vienna, Vienna, Austria.

With interest, we read the article by Lopes et al. about a 16-month-old female with Leigh syndrome (LS) due to the mitochondrial DNA (mtDNA) variant m.8993T>C in the *ATP6* gene with a heteroplasmy rate of >90%.^1^ The patient initially presented axial hypotonia, which markedly increased after anesthesia for a cerebral magnetic resonance imaging (MRI). During follow-up, LS progressed to recurrent exhaustion, lactic acidosis, epilepsy, ataxia, and dystonia.^1^ We have the following comments and concerns.

The key message of the report, that the patient experienced an increase of hypotonia after anesthesia, needs to be more broadly discussed. We should be informed if the patient received local, regional, or general anesthesia. If general anesthesia was applied, we should have been informed which drugs were used in which sequence, and which dosage. It is well known that some anesthetics may worsen the phenotype of a mitochondrial disorder.^2^

It is not comprehensible why the patient received phenytoin (PHT) without having seizures. The patient initially neither had convulsive nor non-convulsive seizures, and the electroencephalography (EEG) did not show epileptic activity. Apnoea and cyanosis one day after anesthesia do not necessarily reflect epileptic activity, but could be also attributed to metabolic, respiratory or cardiac dysfunction. Thus, we should have been informed about the results of the troponin, pro-brain natriuretic peptide (pro-BNP), D-dimer, blood gas analysis, the cardiologic exam, the electrocardiogram (ECG), and the pulmonary investigations during this episode. Furthermore, PHT has been reported to be potentially mitochondrion-toxic,^3^ why it should not be applied as first-line treatment of seizures in mitochondrial disorder (MID) patients. The patient was later switched on phenobarbital and levetiracetam, with beneficial effects, but it was not mentioned if any side effects have occurred. This is crucial as phenobarbital can be mitochondrion-toxic as well,^3^ and may worsen epilepsy. We should also be informed if the ketogenic diet was offered since it has been reported that it can be highly beneficial for mitochondrial epilepsy.^4^

A further shortcoming is that the report does not mention if the mother who carried the mutation with a heteroplasmy rate of 75% presented any phenotypic features of a MID. Despite a lower heteroplasmy rate than her daughter’s, it is conceivable that she had developed clinical manifestations of the mutation as well. Thus, it is recommended that the mother should be prospectively investigated for clinical or subclinical manifestations of a MID.

Overall, this interesting case requires a broader discussion of the adverse reaction to anesthesia, a thorough discussion of the effects of the potentially mitochondrion-toxic antiepileptic regimen, and more widespread discussion of the clinical presentation of the index case shortly after anesthesia. The reaction to anesthesia is crucial as some MIDs manifest only mildly or remain subclinical, considering adverse reactions to anesthesia can be the initial manifestation of the disease.
