# Supplementary material for: ADVERSE REACTION TO ANESTHESIA IN A M.8993T>C CARRIER WITH LEIGH SYNDROME
Source: Rev Paul Pediatr. 2019 Jan-Mar;37(1):135–6. doi: 10.1590/1984-0462/;2019;37;1;00020 (PMC6362374; doi:10.1590/1984-0462/;2019;37;1;00020)
Supplement: Related texts

# Reply: LEIGH SYNDROME: A CASE REPORT WITH A MITOCHONDRIAL DNA MUTATION

**Authors:** Anabela Oliveira Bandeira

**Affiliations:** a Centro Hospitalar Universitário do Porto, Porto, Portugal.

The child was initially followed in a small local hospital, where she was monitored due to a psychomotor developmental delay. In this local hospital, it is not possible to perform MRI, so it was done in a private center. Instructions to parents included a six-hour fasting before the exam. No pre-medication was given, and the MRI was performed with sevoflurane sedation (in 100% oxygen). The child was observed for some time until she woke up, and then she was fed and went home.

Until the performance of the MRI, the hypothesis of mitochondrial disease was not considered as a possible diagnosis, so no special measures were taken. In the hospital where I work, patients with mitochondrial diseases or any other inborn errors of metabolism have anesthetic consultation prior to any intervention. There is an anesthesia protocol for all patients with metabolic diseases.

When the child visited the pediatric care center with apnea and cyanosis, the pediatrician interpreted her case as a convulsive episode. The protocol was diazepam and then phenytoin. No cardiac evaluation, troponin, pro-BNP, D-dimer, or pulmonary investigation was made at that time. After the result of the MRI (Leigh syndrome), one week later, she was referred to our center for the metabolic evaluation. At this time, she was examined by a neuropediatrician and the antiepileptics drugs were changed. The patient had no reaction to phenobarbital. She is still being treated with levetiracetam and phenobarbital.

All patients with mitochondrial disease undergo a cardiac evaluation, which was performed in this patient. The electrocardiography and the echocardiography were normal.

The mother was examined by the adult metabolic team, and she has no symptoms so far.

The effects of using anesthesia in patients with mitochondrial disease have been well reviewed in the literature since almost all anesthetics impair the mitochondrial function^1^ ^,^ ^2^ ^,^ ^3^. The difficulty in this clinical case was that the differential diagnosis did not consider mitochondrial disease as a hypothesis.

All circumstances that can cause metabolic stress should be avoided due to the risk of decompensation in patients with mitochondrial disease. These circumstances include prolonged fasting, hypoglycemia, postoperative nausea and vomiting, hypothermia (with resulting shivering), prolonged tourniquets, acidosis, and hypovolemia.

## References

[1] Niezgoda J, Morgan PG (2013). Anesthetic considerations in patients with mitochondrial defects. Paediatr Anaesth.

[2] Hsieh VC, Krane EJ, Morgan PG (2017). Mitochondrial disease and anesthesia. JIEMS.

[3] Kishikawa J-I, Inoue Y, Fujikawa M, Nishimura K, Nakanishi A, Tanabe T (2018). General anesthetics cause mitochondrial dysfunction and reduction of intracellular ATP levels. PLoS ONE.

# Reply: SÍNDROME DE LEIGH: A PROPÓSITO DE UM CASO CLÍNICO COM MUTAÇÃO NO DNA MITOCONDRIAL

**Authors:** Anabela Oliveira Bandeira

**Affiliations:** a Centro Hospitalar Universitário do Porto, Porto, Portugal.

O seguimento inicial desta criança ocorreu em um pequeno hospital local, onde era seguida por apresentar atraso no desenvolvimento psico motor. Neste hospital local, não é possível fazer RM, assim, ela foi realizada em uma instituição privada. As orientações aos pais incluíam um jejum de seis horas antes do exame. Nenhuma medicação prévia foi administrada e a RM foi realizada sob sedação com sevoflurano (em 100% de oxigênio). A criança ficou em observação por algum tempo até acordar e, em seguida, alimentou-se e foi para casa.

Até o momento da realização da RMN, a hipótese de se tratar de uma doença mitocondrial não foi considerada como possível diagnóstico, portanto, não foram tomadas medidas especiais. No hospital onde trabalho, os pacientes com doença mitocondrial ou qualquer outro erro inato de metabolismo fazem uma avaliação anestésica antes de qualquer intervenção ou procedimento. Há um protocolo de anestesia para todos os pacientes com doenças metabólicas.

Quando a criança deu entrada no serviço de urgência com apneia e cianose, o pediatra interpretou como um episódio convulsivo. O protocolo foi diazepam seguido de fenitoína. Nenhuma avaliação cardíaca, troponina, pró-BNP, dímero D ou investigação pulmonar foi feita naquela ocasião. Após o resultado da RM (síndrome de Leigh) na semana seguinte, ela foi encaminhada para a nossa unidade para avaliação metabólica. Neste momento, ela foi examinada por um neuropediatra e os antiepilépticos foram substituídos. A paciente não teve reação ao fenobarbital. Ela continua sob terapêutica com levetiracetam e fenobarbital.

Todos os pacientes com doença mitocondrial passam por uma avaliação cardíaca, o que ocorreu com esta paciente. O eletrocardiograma e ecocardiograma estavam normais.

A mãe foi avaliada pela equipa de doenças metabólicas dos adultos e não apresenta sintomas até ao momento.

Os efeitos da utilização de anestesia em pacientes com doença mitocondrial estão bem revistos na literatura, praticamente todos os anestésicos enfraquecem a função mitocondrial^1^ ^,^ ^2^ ^,^ ^3^. A dificuldade neste caso clínico foi que no diagnóstico diferencial não foi considerada a hipótese de doença mitocondrial.

Todas as situações que possam causar stress metabólico devem ser evitadas pelo risco de descompensação nos doentes com citopatia mitocondrial. Estas situações incluem jejum prolongado, hipoglicemia, náuseas e vômitos no pós-operatório, hipotermia (com tremor resultante), torniquetes prolongados, acidose e hipovolemia.
